# Automated Method to Determine Two Critical Growth Stages of Wheat: Heading and Flowering

**DOI:** 10.3389/fpls.2017.00252

**Published:** 2017-02-27

**Authors:** Pouria Sadeghi-Tehran, Kasra Sabermanesh, Nicolas Virlet, Malcolm J. Hawkesford

**Affiliations:** Department of Plant Biology and Crop Sciences, Rothamsted ResearchHarpenden, UK

**Keywords:** image categorization, computer vision in agriculture, automated field phenotyping, automated growth stage observation, Field Scanalyzer, wheat heading stage, wheat flowering time

## Abstract

Recording growth stage information is an important aspect of precision agriculture, crop breeding and phenotyping. In practice, crop growth stage is still primarily monitored by-eye, which is not only laborious and time-consuming, but also subjective and error-prone. The application of computer vision on digital images offers a high-throughput and non-invasive alternative to manual observations and its use in agriculture and high-throughput phenotyping is increasing. This paper presents an automated method to detect wheat heading and flowering stages, which uses the application of computer vision on digital images. The bag-of-visual-word technique is used to identify the growth stage during heading and flowering within digital images. Scale invariant feature transformation feature extraction technique is used for lower level feature extraction; subsequently, local linear constraint coding and spatial pyramid matching are developed in the mid-level representation stage. At the end, support vector machine classification is used to train and test the data samples. The method outperformed existing algorithms, having yielded 95.24, 97.79, 99.59% at early, medium and late stages of heading, respectively and 85.45% accuracy for flowering detection. The results also illustrate that the proposed method is robust enough to handle complex environmental changes (illumination, occlusion). Although the proposed method is applied only on identifying growth stage in wheat, there is potential for application to other crops and categorization concepts, such as disease classification.

## 1. Introduction

An estimated doubling in required crop production is projected by 2,050 in order to meet the demand of the rapid growth human population (Tilman et al., 2011). To achieve this, an approximate 38% increase over current increases in annual crop production rates is required, and on not much more arable land. Further concerns exist around not only achieving this target in a changing climate, but also achieving it sustainably, whereby reducing agricultural inputs to reduce the environmental degradation caused by our agricultural footprint (Tester and Langridge, 2010). With wheat providing ≥20% of the worlds calorie and protein intake (Braun et al., 2010), the requirement to increase yield and production is widely recognized.

Breeding and precision agriculture, including information-based management of agricultural systems, are fundamental for achieving sustainable increases in wheat productivity and production. One component critical to both crop breeding and precision agriculture is the monitoring of developmental growth stages, as (i) it helps crop producers understand which phases of wheat development are most vulnerable to biotic and environmental stresses, and (ii) supports precision agriculture by helping making informed-decisions around which treatment should be applied, to what location and when to apply it. Two critical growth stages monitored in crops, including wheat, are heading date and flowering time, as cultivars with appropriate heading time to their target environment and life cycle duration will help maximize yield potential (Snape et al., 2001; Zhang et al., 2008).

The monitoring of heading and flowering stages are still primarily performed by human eye, which is labor-intensive and time-consuming, as these observations need to be performed on up to thousands of cultivars/varieties on a daily or bi-daily basis, given the importance in catching the starting date of these growth stages. Given that manual growth stage monitoring is also subjective, different observers may likely perceive the growth stage of the same plot differently, which introduces human-error into obtained data.

Computer vision offers an effective alternative for growth stage monitoring because of its low-cost (relative to man-hours invested in to manual observations) and the requirement for minimal human intervention. Computer vision has facilitated automation in high-throughput phenotyping, as well as areas of agriculture, such as disease detection (Pourreza et al., 2015), weed identification (Guerrero et al., 2012) and quality control (Alahi et al., 2012; Valiente-González et al., 2014). Despite the efforts of computer vision specialists over the past decades, developing reliable image-based model to identify and categorize images based on visual information is still difficult to achieve and remains an unsolved problem in the computer vision community. The visual recognition of object categories is a natural and trivial task for humans. Humans can recognize objects effortlessly even with changes in an object's appearance, such as viewing direction or a shadow being cast across the object. On the other hand, in computer vision it can be a challenging task to achieve such level of performance due to the difficulties inherent in the problem. Images are quite abstract and subjected to illumination, scale, deformation, background clutter, etc. Moreover, in computer vision, teaching a machine to distinguish and categorize objects is all about teaching it which differences in the image is matters and which don't, by scanning through diverse datasets, which is a computationally exhaustive process.

Computer vision has shown promise in detecting growth stages of crops. For seedling emergence, color segmentation approaches have been applied in maize (Yihang et al., 2014) and oilseed rape (Yu et al., 2013), using images acquired from a digital camera. Some approaches for observing later growth stages, such as heading date and flowering stage, have also been developed. Zhu et al. (2016), developed a method to detect wheat heading stage from RGB images using a two-step coarse-to-fine detection approach. For flowering stages, Guo et al. (2015) used object-recognition to detect flowering stages from rice panicles. Although the approaches by Zhu et al. (2016) and Guo et al. (2015) were effective on a single variety, within small patches of whole canopies, applications that are more versatile and that also can be applied on different varieties on larger scale canopies are required.

This study utilizes a novel visual-based approach to monitor heading and flowering stage of field grown wheat, through the automated learning of the visual consistency between classes of canopy images, in order to identify the critical growing stages of wheat (e.g., whether ears are emerging in canopies). This method searches through an image database to identify and retrieve images containing emerged wheat ears and ears at flowering stages. This visual-based approach is:

- Not limited to specific wheat cultivars and is applicable to a variety of categorical wheat without implementing specific tuning for each category.
- Robust to handle illumination changes and natural lightning conditions in the field.
- Robust in distinguishing the early emerged ears, despite the color difference between ears and leaves being hardly distinguishable to the naked eye.

## 2. Materials and methodology

**Figure 1:**
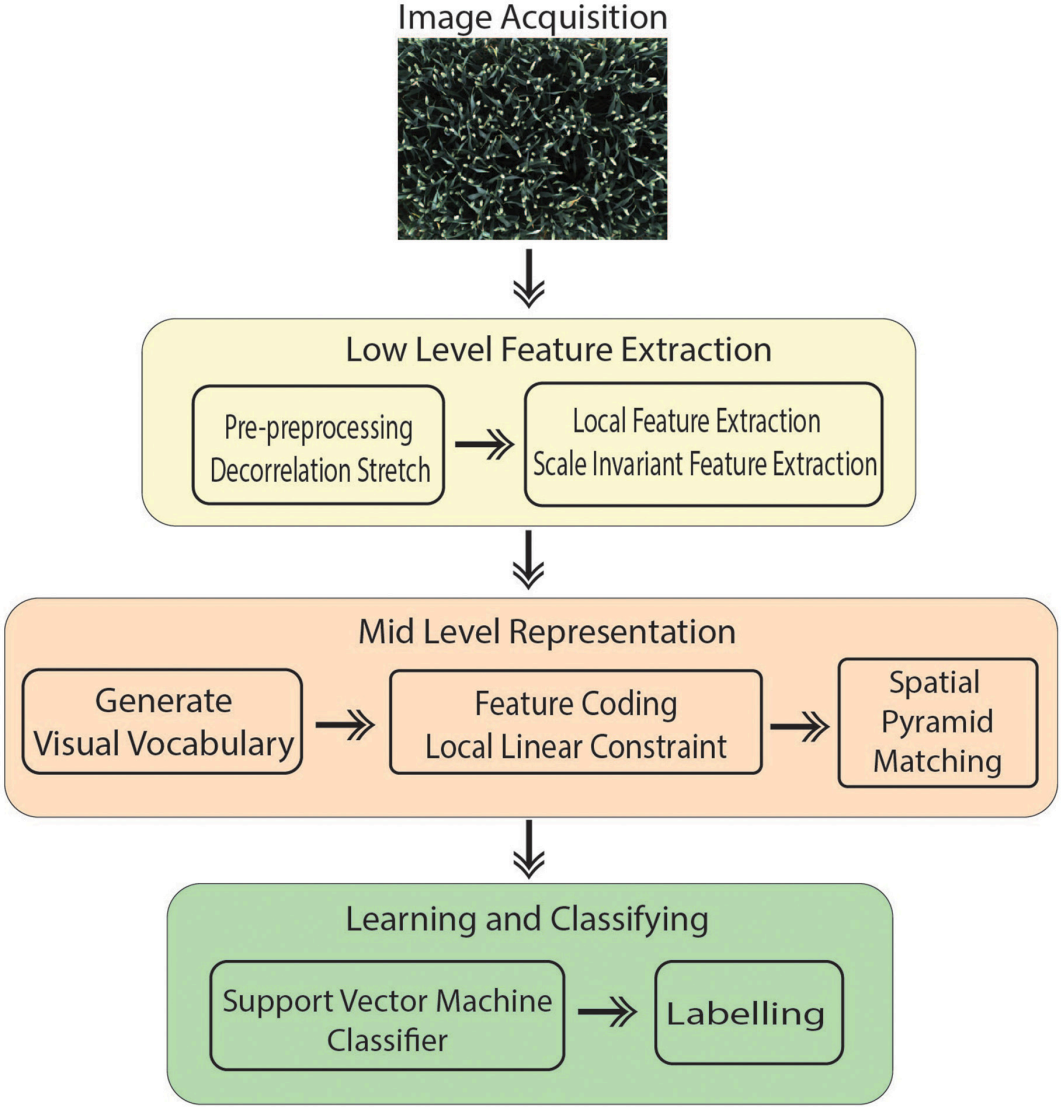
**Schematic representation of the method**.

The introduced technique is performed in four main steps (Figure 1):

- Image acquisition: A RGB image is captured from 8 MP camera mounted inside the camera bay.
- Pre-processing of the images to improve the contrast.
- Extracting features that contain suitable information to discriminate images at the category level.
- Classification: Images classified in different categories as specified.

Bag of Visual Words (BoVW) proved to be the leading strategy in computer vision applications such as image retrieval and image categorization (Csurka et al., 2004); thus, it is being opted for the presented work. Categorizing digital images, embarks on extracting features and creating a visual vocabulary for the given dataset. It comprises of following states:

1. Extracting features.
2. Constructing visual vocabulary by clustering.
3. Using multi-class classifier for training using bags as feature vector.
4. For the testing image, obtain the nearest vocabulary based on the most optimum prediction of classifier.

However, in this study, several steps are integrated in the process to improve the overall performance compared to Csurka et al. (2004) described in Section 2.3. Our method treats canopy images acquired automatically in the field as a collection of unordered appearance descriptors extracted from local patches; then, quantizes them into discrete visual words. Each image is defined by a feature vector listing the number of regions which belongs to each cluster and are later used to train a classifier. In addition, the location information is taken into account which is one of the important factors in object recognition scenarios. In the final step, a linear Support Vector Machine (SVM) classifier is used to determine pre-defined classes (e.g., ear emergence, flowering). The experimental results show that the introduced method is capable of automatically identifying key wheat growing stage with high accuracy and efficiency (Section 3).

### 2.1. Field experiment and image acquisition

**Figure 2:**
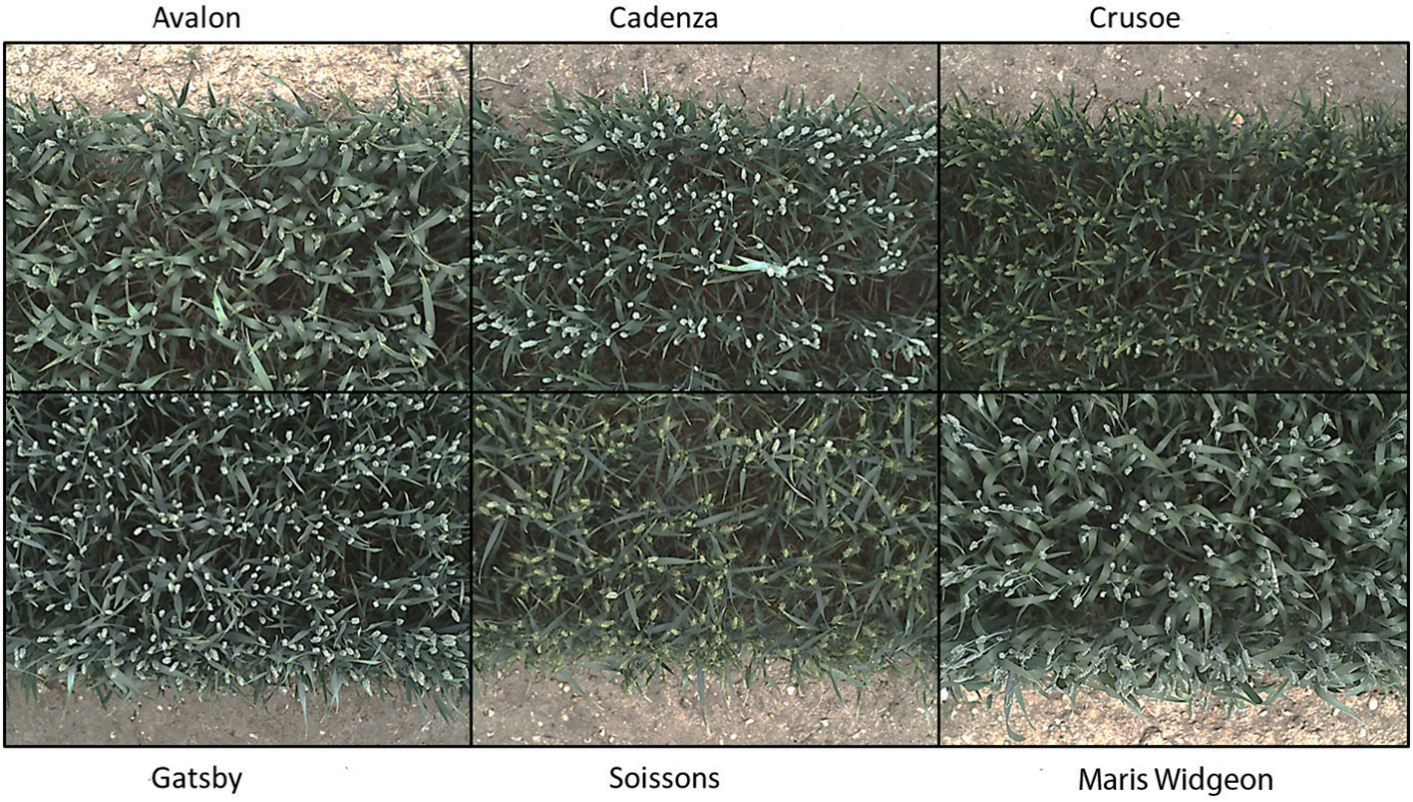
**Digital images of the six contrasting wheat cultivars (***Triticum aestivum*** L. cv. Avalon, Cadenza, Crusoe, Gatsby, Soissons, and Maris Widgeon) used, at growth stage Z5.9**.

Six wheat cultivars (*Triticum aestivum* L. cv. Avalon, Cadenza, Crusoe, Gatsby, Soissons and Maris Widgeon) were grown in the field at Rothamsted Research, Harpenden, UK, sown in Autumn 2015 and maturing in 2016. These cultivars were selected as they had different properties visible to the naked-eye (awns/no awns, differing wax properties, straight/floppy leaves, different ear morphology) (Figure 2). All cultivars were sown 20 October 2015, at a planting density of 350 plants/*m*^2^. Nitrogen (N) treatments were applied as ammonium nitrate in the spring, at rates of 0 kg *ha*^−1^ (residual soil N; N1), 100 kg *ha*^−1^ (N2) and 200 kg *ha*^−1^ (N3) (Figure 4).

**Figure 3:**
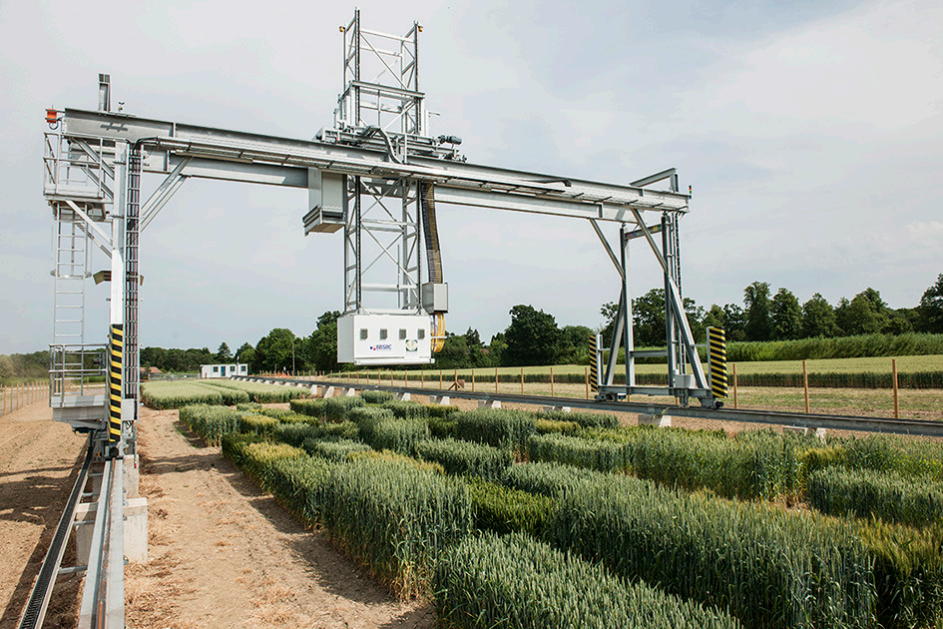
**The Field Scanalyzer at Rothamsted research**.

The Field Scanalyzer phenotyping platform (LemnaTec GmbH; Virlet et al., 2017) was used to acquire all images (Figure 3). The Field Scanalyzer is a fully-automated, high-throughput, fixed-field phenotyping platform, carrying multiple sensors for non-invasive monitoring of plant growth, morphology, physiology and health. The on-board visible camera (color 12 bit Prosilica GT3300) was used to acquire RGB images at high-resolution (3,296 × 2,472 pixels). The camera is positioned perpendicular to the ground, and automatically adjusts to ensure a 2.5 m distance is maintained between the camera and canopy. The camera is set up in auto-exposure mode, to compensate for outdoor light changes. Wheat canopies were imaged daily during three stages of ear emergence: Stage 1 (Zadoks scale Z5.0; 3–5 June 2016 Zadoks et al., 1974); Stage 2 (Z5.3–Z5.7; 7–10 June 2016) and Stage 3 (≥ Z5.9; 12–14 June 2016), as well as flowering stage (14–18 June 2016). In addition, illumination conditions were recorded during the image acquisition (Table 1). Manual growth stages were recorded daily or on alternating days during heading and flowering. The growth stage of the plot was defined manually by the stage of ≥50% of the plot. Videos and more information of the Field Scanalyzer platform can be accessed in our website: http://www.rothamsted.ac.uk/field-scanalyzer.

**Figure 4:**
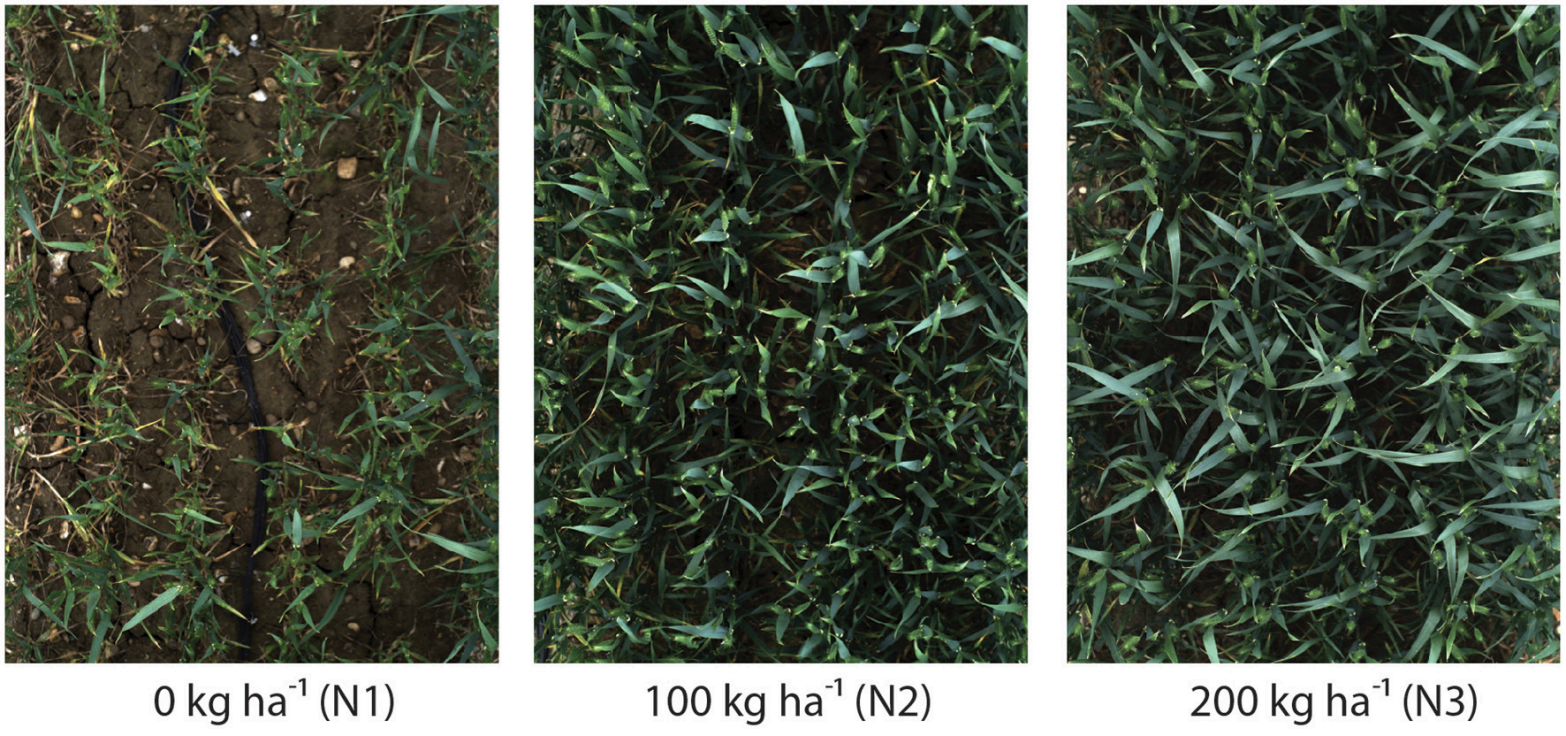
**Digital images highlighting the impact of 0 kg ***ha***^**−1**^ (N1), 100 kg ***ha***^**−1**^ (N2) or 200 kg ***ha***^**−1**^ (N3) nitrogen fertilizer application on canopy complexity**. Images were acquired 2.5 m above *Triticum aestivum* L. cv. Soissons canopies.

**Table 1:** **Date, start/end time, and PAR values of each images acquisition periods during ears emergence and flowering stages**.

**Date**	**Start**	**End**	**PAR (*μmol.m*^−2^.*s*^−1^)**
03/06/2016	11:19:16	12:08:24	404 ± 58
04/06/2016	11:53:11	12:42:41	512 ± 67
05/06/2016	08:29:34	09:18:38	287 ± 43
07/06/2016	13:33:40	14:25:07	1,037 ± 95
08/06/2016	08:07:05	08:58:46	315 ± 19
08/06/2016	18:05:53	18:38:33	528 ± 180
09/06/2016	08:32:25	09:24:05	530 ± 215
10/06/2016	07:43:46	08:37:33	461 ± 33
12/06/2016	14:32:33	15:26:18	703 ± 304
13/06/2016	09:49:04	10:41:12	555 ± 110
14/06/2016	10:14:16	11:06:10	800 ± 196
14/06/2016	15:01:19	15:33:55	569 ± 121
16/06/2016	08:02:55	08:35:57	363 ± 31
18/06/2016	10:51:31	11:44:17	919 ± 238

*PAR mean and standard deviation values are computed from the 54 scans collected during one acquisition periods*.

### 2.2. Image pre-processing and enhancement

**Figure 5:**
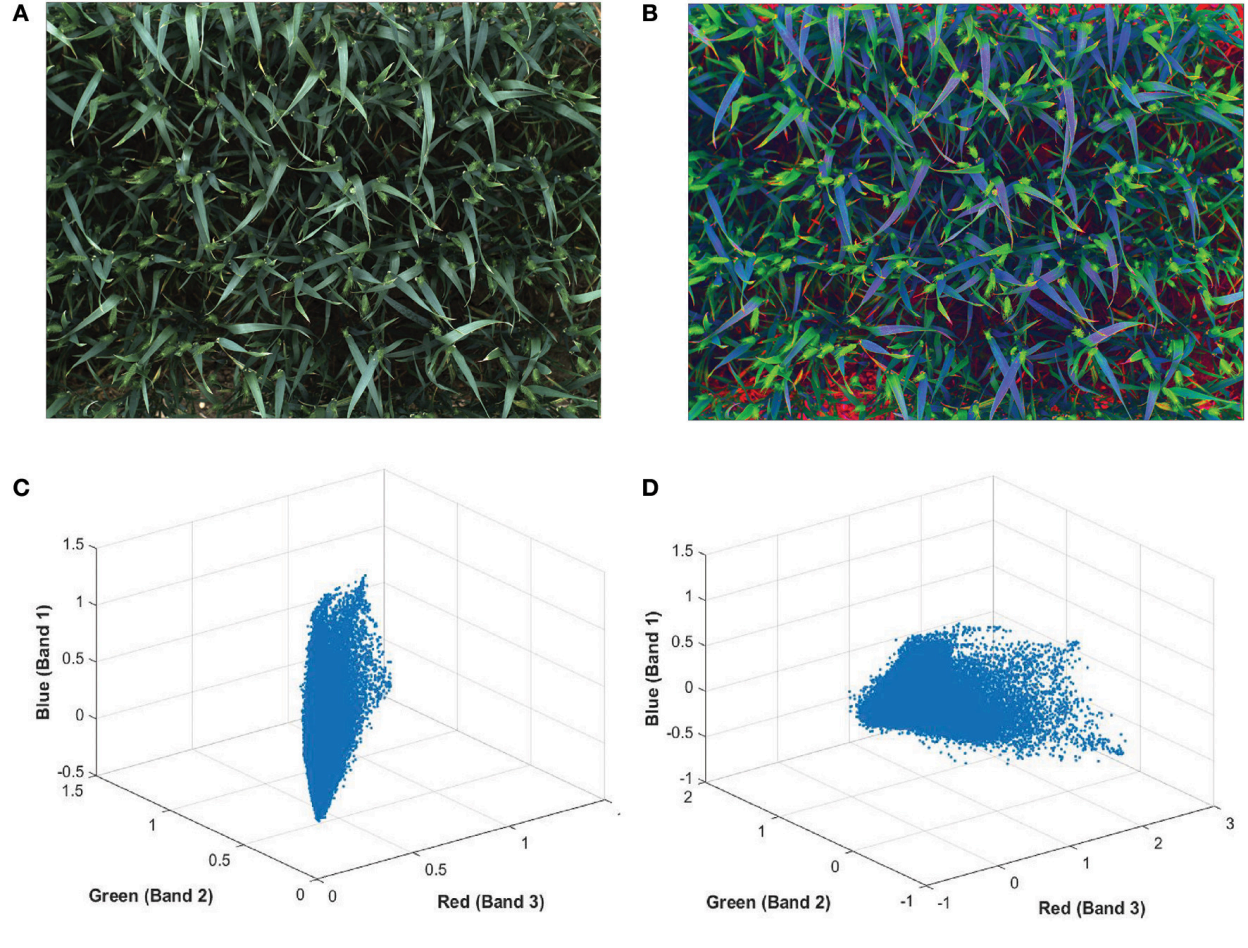
**Digital image of wheat (***Triticum aestivum*** L. cv. Soissons) canopy (A)** before and **(B)** after enhancement of image contrast and application of decorrelation stretching. Scatterplot of every pixels normalized red, green blue (RGB) values from **(C)** the original image and **(D)** after applying the decorrelation stretch and contrast increase.

The color of ears at early development stages are very similar to leaves and hardly discernable with the naked-eye (Figures 5A,C). In order to make the ears stand out in canopies and discriminate them from the background more easily, a pre-processing method is applied on plot images before extracting features, known as decorrelation stretching (DS). The decorrelation stretching technique enhances the color differences and increasing the image contrast in each plot image by removing the inter-channel correlation found in the pixels (Gillespie et al., 1986). Therefore, it allows to see details such as ears that are otherwise too subtle for the naked-eye (Figures 5B,D). If the red, green, and blue values of pixels are treated coordinates in space, decorrelation stretch moves these points in space further apart, so they become much easier to see a difference between them.

The DS among the RGB channels is achieved through principle component analysis (PCA) to remove inter-channel correlation in an image. The application of PCA to the digital analysis of an image is based on first, calculating the covariance matrix between the three RGB bands. Then, obtaining eigenvectors and eigenvalues. Finally, rotating the original image vector to a new space by multiplying it by the eigenvectors (Equation 1) (Jolliffe, 2002; Cerrillo-Cuenca and Sepúlveda, 2015).

(1) $$\begin{array}{l} p_{n}=R^{T}i_{n} \end{array}$$

where *i*_*n*_ is the image vector; *n* is the number of pixels; and *R* is the rotation matrix.

Campbell (1996) proposed a general framework consists of the following steps:

- Calculating *p*_*n*_ from Equation (1), eigenvalues and eigenvectors are obtained from the correlation matrix or alternatively from the covariance matrix.
- Generating a stretch vector: diagonalize the covariance matrix composed by the inverse of the eigenvectors:
  (2) $$\begin{array}{l} D=\left[\begin{array}{ccc} \frac{1}{\sqrt{v_{1}}} & 0 & 0\\ 0 & \frac{1}{\sqrt{v_{2}}} & 0\\ 0 & 0 & \frac{1}{\sqrt{v_{3}}} \end{array}\right] \end{array}$$
  where *D* is a diagonal matrix; *v* denotes each of the eigenvalues. Alternatively, *D* can be multiplied by an integer value that serves to achieve a higher contrast in the image (Alley, 1996). Finally, the resultant matrix is applied to *p*_*n*_ (Equation 3). At this step, the matrix is re-centered and stretched its values to a maximum.
  (3) $$\begin{array}{l} w_{n}=Dp_{n} \end{array}$$
- The inverse transform is applied to map the colors back to the original space. The information is decorrelated into a new vector *c*_*n*_ composed of three matrices (RGB) (Equation 4)
  (4) $$\begin{array}{l} c_{n}=Rw_{n}=RDR^{T}i_{n} \end{array}$$
- Finally, a standard deviation value is applied to visually increase the contrast (Alley, 1996).

### 2.3. Bag of visual words construction

The first step of BoVW framework corresponds to feature extraction. Fixed length feature extraction techniques based on color (Swain and Ballard, 1991; Chen et al., 2010), texture (Duda et al., 2000), shape (Mehrotra and Gary, 1995), or a combination of two or more techniques, extract pixel values of an image only. These are excellent in comparing the overall image similarity (Angelov and Sadeghi-Tehran, 2016); however, they are not scale or rotation invariant. Moreover, they are very sensitive to noise and illumination changes; thus, are unable to describe the object-based properties of the image content.

As opposed to global feature extraction methods mentioned earlier, local extraction algorithms are robust to partial visibility and clutter. It is an ideal candidate for object recognition, template matching and image mosaicing. There are several feature detector methods, which are scale and rotation invariant. They are also robust enough to handle illumination changes and resistant to geometry (Bay et al., 2006; Leutenegger et al., 2011; Alahi et al., 2012). Among the proposed descriptors, Scale Invariant Feature Transform (SIFT) is selected due to its excellent performance attested in various applications (Mikolajczyk and Schmid, 2005). It returns an *N* × 128 dimension image descriptor, where *N* is the number of features.

**Figure 6:**
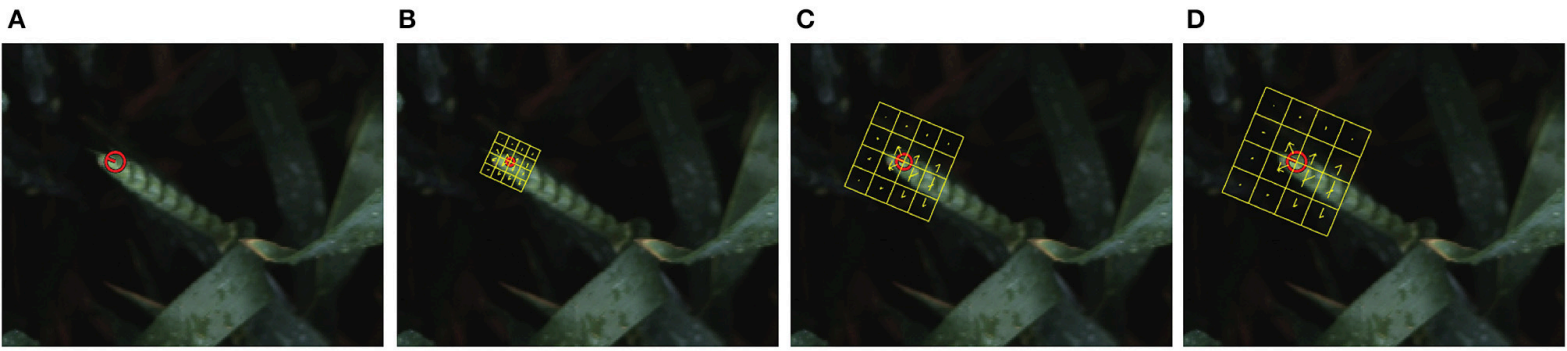
**(A)** A single keypoint candidate in the image; **(B–D)** SIFT descriptor calculated at different scales of 4, 8, and 10; At each scale, the descriptor has 4 × 4 patches (color coded in yellow), which are rotated to the dominant orientation of the feature point. Each patch is represented in gradient magnitudes of eight directions, represented by yellow arrows inside each bin.

SIFT consists of Lowe (2004):

- **Constructing a scale space**: in this stage, location and scales of each keypoint are identified. Laplacian-of-Gaussian (LoG) is calculated for an image with various σ. Due to change in σ, LoG detects blobs of various sizes, then the local maxima can be found across the scale and space with a list of (*x, y*, σ) values, which show there is a candidate keypoint at location *x, y* with scale of σ. However, in order to reduce the computational complexity, SIFT uses Difference-of-Gaussian (DoG) which is a convolved image in scale space separated by a constant factor *k*:
  (5) $$\begin{array}{r} D\left(x,y,\sigma \right)=\left(G\left(x,y,k\sigma \right)-G\left(x,y,\sigma \right)\right)*I\left(x,y\right)\\ =L\left(x,y,k\sigma \right)-L\left(x,y,\sigma \right) \end{array}$$
  where *I*(*x, y*) is an input image; *L*(*x, y, kσ*) is the scale space of an image; *G*(*x, y, kσ*) is variable-scale Gaussian.
  *D* is computed by simple image subtraction and the Guassian image is sub-sampled by a factor of 2 and produces DoG for the sampled image. Once the DoG is computed, images are searched for local extrema over space and scale. For instance, one pixel is compared with its *n* × *n* neighborhood (*n* = 3 in our experiment) as well as 9 pixels in the next scale and 9 pixels in previous scales (Lowe, 2004).
- **Keypoint localization and filtering**: Once the location of keypoints candidates are found, they are refined and some are eliminated to get a more accurate location of extrema. For instance, if the intensity at the extrema is less than a certain threshold (threshold <0.03) it is rejected. In addition, edges and low contrast regions are considered as bad keypoints and will be rejected.
- **Orientation assignment**: The orientation of each keypoint is obtained based on image gradient and local image gradient directions to achieve rotation invariance. Depending on the scale a neighborhood is taken around the keypoint location and the gradient magnitude and direction is calculated in that region.
- **Keypoint descriptor**: In order to generate a keypoint descriptor, the local image descriptor is computed for each keypoint based on image gradient magnitude and orientation at each image sample point in a region centered at keypoint. These samples build a 3D histogram of gradient location and orientation; with a 4 × 4 array location grid and 8 orientation bins in each sample, which creates 128 element dimensions of the keypoint descriptor, causing robustness against changes in scale and rotation (Figure 6).

**Figure 7:**
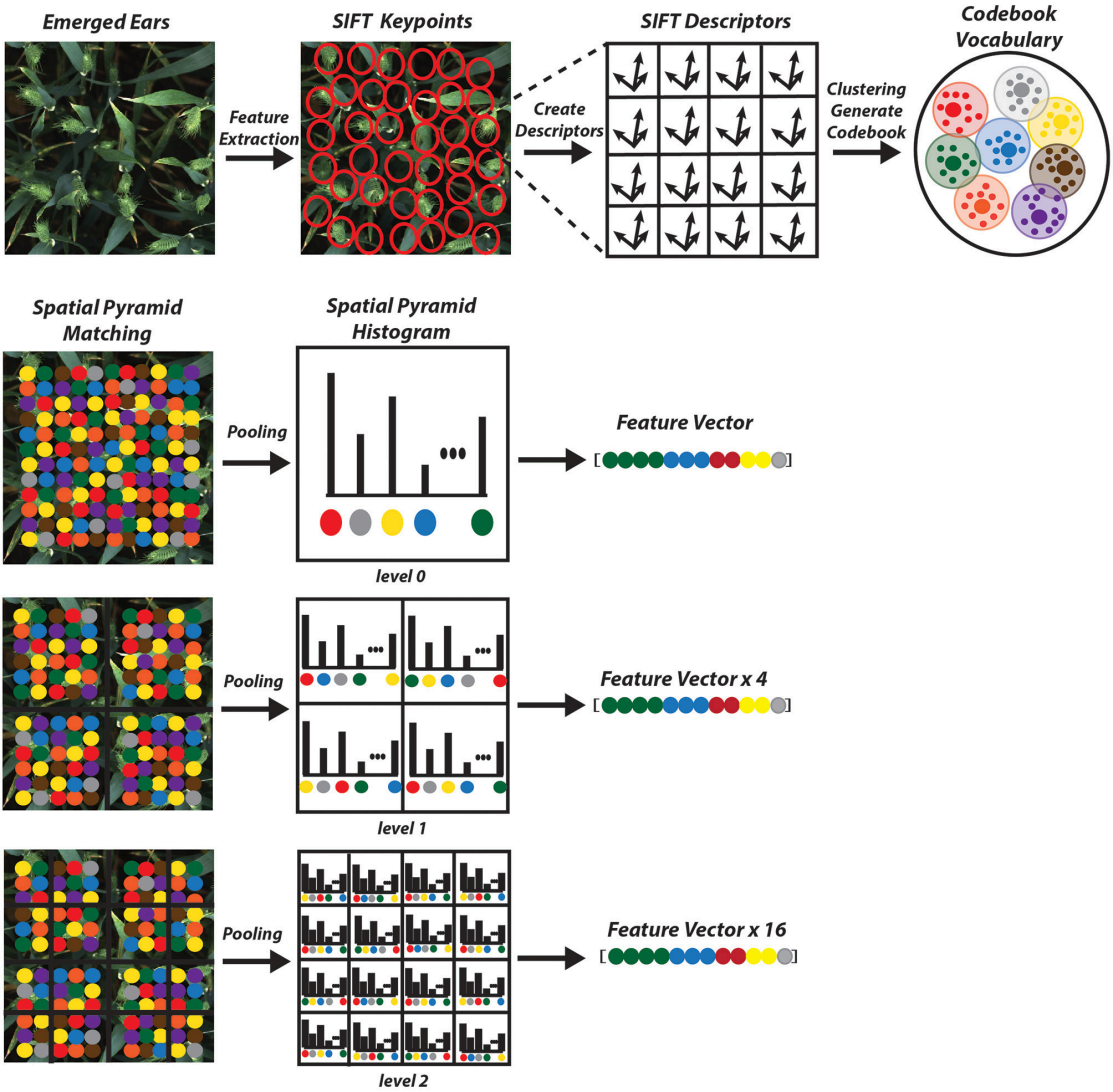
**Schematic representation of the proposed method**.

The next step is to form clusters of similar features and assign them as visual words. The objective of constructing codebook is to relate features of testing images to the features previously extracted from the training image samples (Figure 7). Although in the field of unsupervised learning, clustering is a standard procedure, there is no single clustering algorithm that can be applied uniformly to all the application domains or address all related issues in a satisfactory manner. Here, a partition-based clustering approach known as *K*-means clustering is used to quantize each descriptor and generate a codebook. The process is iterative as follows (Lloyd, 1982):

**Algorithm 1:** *K*-means clustering procedure

1: Select *K* points as initial centers
2: **repeat**
3: Assign each input data to its closest center
4: Re-compute the center of each cluster by averaging all the members in the clusters
5: **until**
6: convergence which means no pixel shifts from one cluster to another; centers do not change

In *K*-means the number of clusters is pre-defined beforehand and it should be large enough to identify relevant changes in each wheat cultivars. For an image having *N* features, the model will distribute the features with *K* clusters, which is the size of the visual vocabulary. We have been able to find the optimum numbers and get very good results with number of vocabulary (codebook) *K* = 2000 (Table 2).

**Table 2:** **Comparison of different methods applied on the three heading stages**.

**Decorrelation pre-processing**	**Feature extraction**	**Coding method**	**Spatial pyramid**	**Vocabulary length**	**Accuracy Z5.0 (%)**	**Accuracy Z5.3–Z5.7 (%)**	**Accuracy ≥ Z5.9 (%)**
Yes	SIFT	LLC	Yes	2,000	95.24	97.79	99.59
No	SIFT	LLC	Yes	2,000	57.29	82.20	85.38
Yes	SIFT	k-NN	Yes	2,000	90.54	94.48	96.97
Yes	SIFT	LLC	No	2,000	92.90	96.54	96.90
Yes	SURF	LLC	Yes	2,000	56.84	71.55	78.45
Yes	SIFT	LLC	Yes	1,000	93.91	97.52	98.91
Yes	SIFT	LLC	Yes	1,500	94.61	97.64	99.24
Yes	SIFT	LLC	Yes	2,500	94.90	97.59	99.49

The codebook is used for quantizing features. A vector quantizer takes a feature vector and maps it to the index of the nearest code vector in a codebook. In our work, in order to project the descriptors onto the codebook elements, Local Linear Constraint (LLC) (Wang et al., 2010) is used to generate a final vector which represents an image. LLC reduces the computational complexity to *O*(*K*+*K*) (where *K* is the length of the codebook; *K* = 2000 in this case) for each descriptor and can achieve acceptable image classification accuracy even with a linear SVM classifier (Wang et al., 2010).

The main drawback of BoVW is that it is unable to capture spatial relationships between images. In order to preserve the spatial relations of the code vector Spatial Pyramid Matching (SPM) is implemented where the entire image is divided into levels. Each image is divided into spatial sub-regions and computes histograms of features from each sub-region. Each level divides the image into 2^*l*^ × 2^*l*−1^; where *l* is level (Grauman and Darrell, 2005; Lazebnik et al., 2006). The features are computed locally for each grid and the spatial information is incorporated into histograms. A three level SPM is used with first, level 0 which comprises of a single histogram; level 1, comprising of 4 histograms, finally level 2, comprising of 16 histograms (Figure 7). The histogram from all the sub-region are concatenated together to generate the final representation of the image for classification. The result is a feature weighted histogram of 21 × *K* (number of words = 2000). Using such method will preserve the discriminative power of the descriptors; in addition, changes in the positioning of the objects and variations in the background will not affect the overall performance of the method.

### 2.4. Learning model

The construction of the model for our image annotation is based on the supervised machine learning principle. Supervised learning can be thought as learning by examples represented by a set of training-testing samples. In order to classifying unknown testing images, a certain number of training images are used for each class to train the classifier. A classifier approximates the mapping between the images and correctly labels the training set, called the *training* phase. After the model is trained, it is able to classify unknown image, into one of the learned class labels.

In our model, the complexity of visual categorization is reduced to two-class with positive and negative training patches. The SVM classifier is used as our classifier of choice as it is fast and can handle the long feature vectors generated by the SPM. During the training phase, labeled images (ears and background) are fed to the classifier and used to adapt a statistical decision procedure. Among many available classifiers, linear SVM with Hellinger kernel is used to predict the unlabeled test images and retrieve as much of the data as possible in a high ranked position. Feature vectors generated from each image are normalized to a unit Euclidean norm and used for a linear SVM classifier with the Hellinger kernel to compute the feature map (Vedaldi and Zisserman, 2012).

(6) $$\begin{array}{l} K\left(n,n\prime \right)=\overset{d}{\underset{m=1}{\sum }}\sqrt{n_{m}{n\prime }_{m}};n=\left[n_{1},\ldots ,n_{d}\right];n\prime =\left[n_{1}^{\prime },\ldots ,n_{d}^{\prime }\right] \end{array}$$

where *n* and *n*′ are normalized histograms; *d* = 42, 000

One-vs.-all strategy is chosen to train the SVM. Two classes are trained, each labels the sample inside one class as +1 and other samples (background) as -1. The SVM calculates the similarity of all trained classes and assigned the test image to the class with the highest similarity measure.

## 3. Experimental results and discussion

The experiment is divided into two sections of identifying ear emergence and flowering stages from the digital images acquired in the field. In the first section, ear emergence was tested at different time points, from early stages where only few spikelets are visible, to a more advanced stage where ears are fully emerged (Section 3.1). In the second part of the experiment, the method was tested to identify flowering growth stage during anthesis (Section 3.2). The training dataset for the ear emergence experiment includes images with ears at different emergence stages (positive class) and leaves, soil, etc. (negative class), which are manually cropped and stored in the dataset. On the other hand, the training dataset for the flowering experiment contains ears at different flowering time points (positive class) and ears before and after flowering (negative class). The collected dataset focuses on different challenges regardless of light conditions in the field and to demonstrate the robustness of the method to environmental changes. In addition, the versatility of the proposed technique were also tested by minimizing the number of cultivars as training patches, and evaluating the method on more varieties.

The research was conducted with the following specifications. System comprised of 24 GB RAM, Intel quad core Processor (3.40 GHz) with Windows 10 OS. The models have been developed in MATLAB (Mathworks Inc.); however, to improve the processing time, some of the algorithm, such as SIFT were written in C++ programming language. Utilities like VLFeat library (Vedaldi and Fulkerson, 2010) to extract features as well as LibLinear library (Fan et al., 2008) to train and test the SVM classifier. Using the above configured computer system, extracting features and generating code vectors from each training image approximately takes 0.45 s. However, the processing time increases to 5.4 s for each testing patch with resolution of 3,298 × 2,474 pixels.

Precision (Pr) and Recall (Re) are the most commonly used measurements to evaluate the performance of image retrieval systems. Thus, it is used in our experiment to quantitatively assess the precision of the proposed approach in detecting the two main growing stages of ear emergence and flowering. Precision is defined as the ratio of the number of retrieved relevant images *N*_*r*_ to the total number of retrieved images *N* (Equation 7); on the other hand, Recall is defined as the number of retrieved relevant images *N*_*r*_ over the total number of positive images *N*_*t*_ available in the database. In an ideal scenario, both Pr and Re should have high values (1). Therefore, instead of using Pr and Re individually, usually accuracy curve is used to characterize the performance of the retrieval system.

(7) Pr=NrN;Re=NrNt

### 3.1. Ear emergence

**Figure 8:**
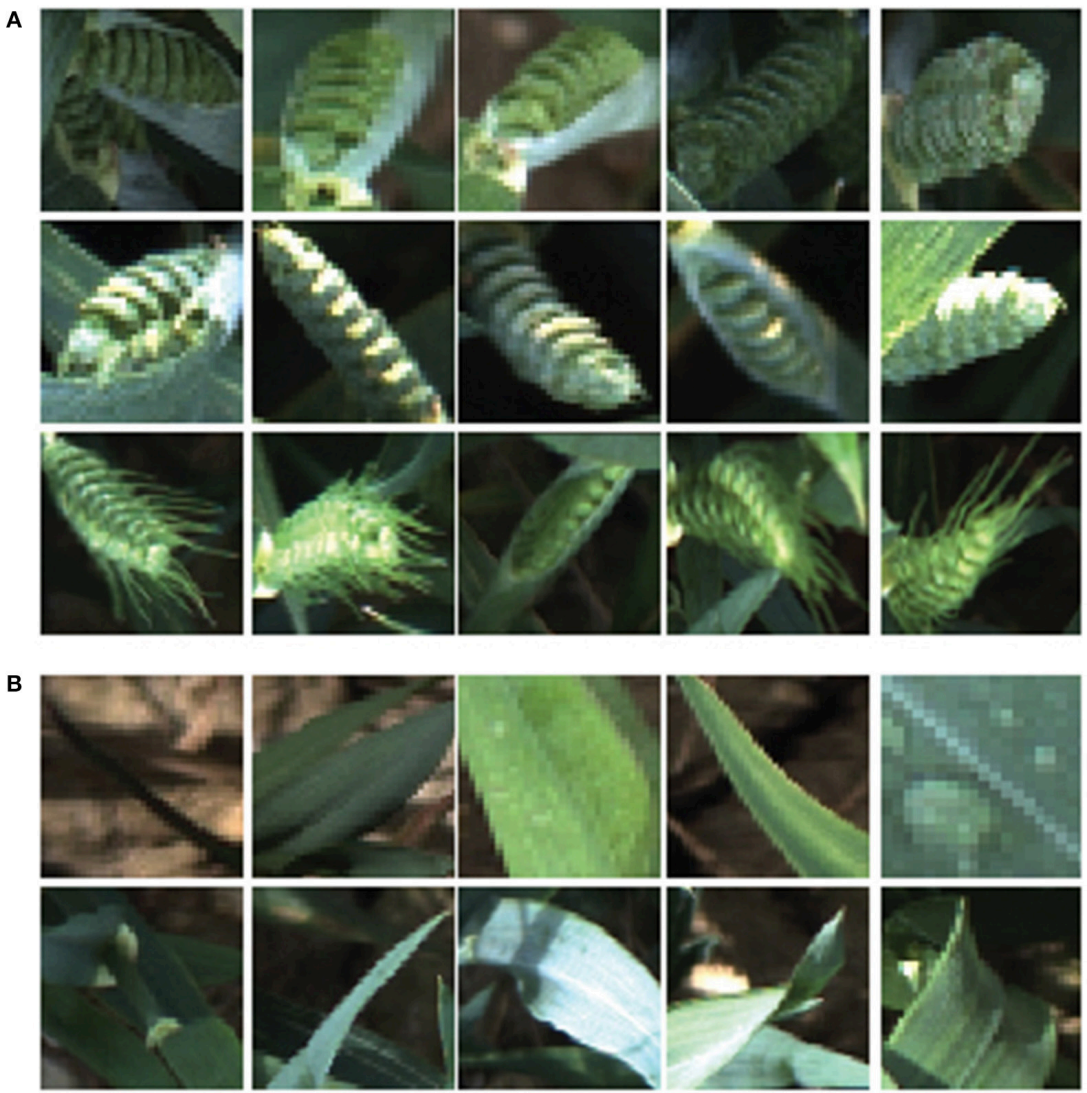
**Example of ear emergence training patches**. Note that the training patches are not necessarily of equal size and resized for illustration purposes. **(A)** Examples of positive training patches of three different wheat cultivars (*Triticum aestivum* L. cv. Soissons, Avalon, and Cadenza). **(B)** Examples of negative training image patches.

The learning process starts with 1,000 training image samples divided into 500 ears (positive class), which are manually cropped from full size canopy image and 500 background images (negative class). Figure 8 shows image samples randomly selected from training patches which are not necessarily the same dimensions. Moreover, to observe the field challenges during data acquisition, ears are selected from different positions and illumination conditions (with or without occlusions and overlapping; sunny or cloudy days). Three different wheat cultivars are used as a training dataset including Avalon, Cadenza, and Soissons. Cadenza can present short awnlettes/scurs at the ear tip, although most of the times no awns are present in contrast to Soissons which is an awned variety. Although three wheat cultivars were used as a training dataset, six cultivars including Maris Widgeon, Avalon, and Gatsby are tested to highlight the versatility of the proposed technique.

Ear identification was evaluated at three different time points of the emergence period, (i) at Z5.0, when the ears start to be visible (first spikelet of inflorescence visible), (ii) between Z5.3–Z5.7, when 1/4 to 3/4 of the ears are emerged and (iii) at Z ≥ 5.9, when ears are fully emerged (Figure 8). Each time point was tested independently from datasets containing 80 images (40 with ears present and 40 without) of full size wheat canopies with the original resolution of 3,298 × 2,474 pixels.

**Figure 9:**
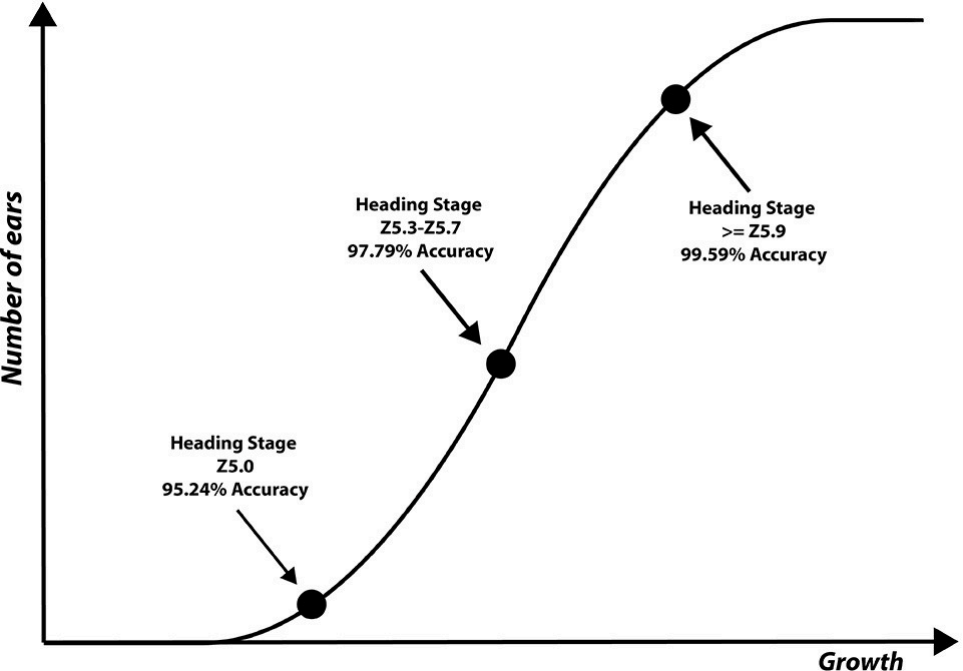
**Three ear development stages visually scored and used to evaluate the performance of the proposed method**.

The results for each ear development stage are shown in Table 2. The accuracy of the method is evaluated using different techniques at different processing stages. (i) presence/absence of decorrelation processing, (ii) SIFT vs. SURF, (iii) LLC vs. KNN, (iv) presence/absence of spatial pyramid and (v) the vocabulary length. As shown in the Tables, the best performance was obtained using decorrelation pre-processing, SIFT, LLC coding, and a 2,000 entry codebook. The best performance at heading stage Z5.0 is 95.24%, and for heading stages Z5.3–Z5.7 and ≥ Z5.9 are 97.79 and 99.59%, respectively (Figure 9). Out of the eight tested scenarios, we achieved accuracy of > 90% at Z5.0 and > 96% at Z5.9 in six scenarios. The impact of codebook size on the performance of the method was also investigated. It is clearly shown that the increasing number of codebook improves the accuracy; however, the accuracy plateaus at 2,000 visual words. Moreover, the low-level feature extraction and the decorrelation pre-processing technique has the biggest influence in the quality of results; especially in the early heading (Z5.0). The main conclusion is that mid-level feature coding and classification are highly impacted by the low level pre-processing and feature extraction techniques.

**Figure 10:**
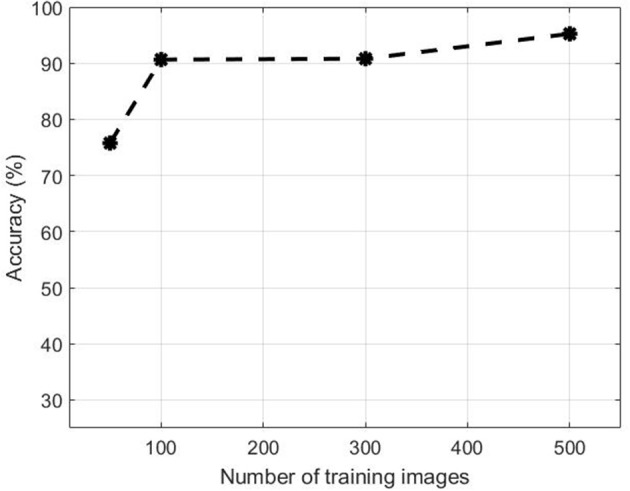
**Accuracy of the proposed method over number of training image samples**.

Figure 10 illustrates the performance evolution of the heading stage Z5.0 over the number of images in the training dataset. for both positive and negative data. Training patches of 50, 100, 300 were selected randomly apart from the full set when all 500 samples were used. The accuracy improves by increasing the number of training samples. The accuracy increased from 75.77 to 90.65% when the training dataset increased from 50 to 100. On the other hand, there was no substantial change in accuracy between 100 and 200 samples. However, the performance jumped by more than 5% from 90.80 to 95.24% when the dataset increased to 500.

### 3.2. Flowering time

Similarly to ear emergence identification, two training classes were created, which comprised of three wheat cultivars (Soissons, Maris Widgeon, and Cadenza). The first class (positive class) contained 140 manually cropped images at flowering stage while the second class (negative class) contained the same number of images as the positive class, but with ears before and after flowering.

**Figure 11:**
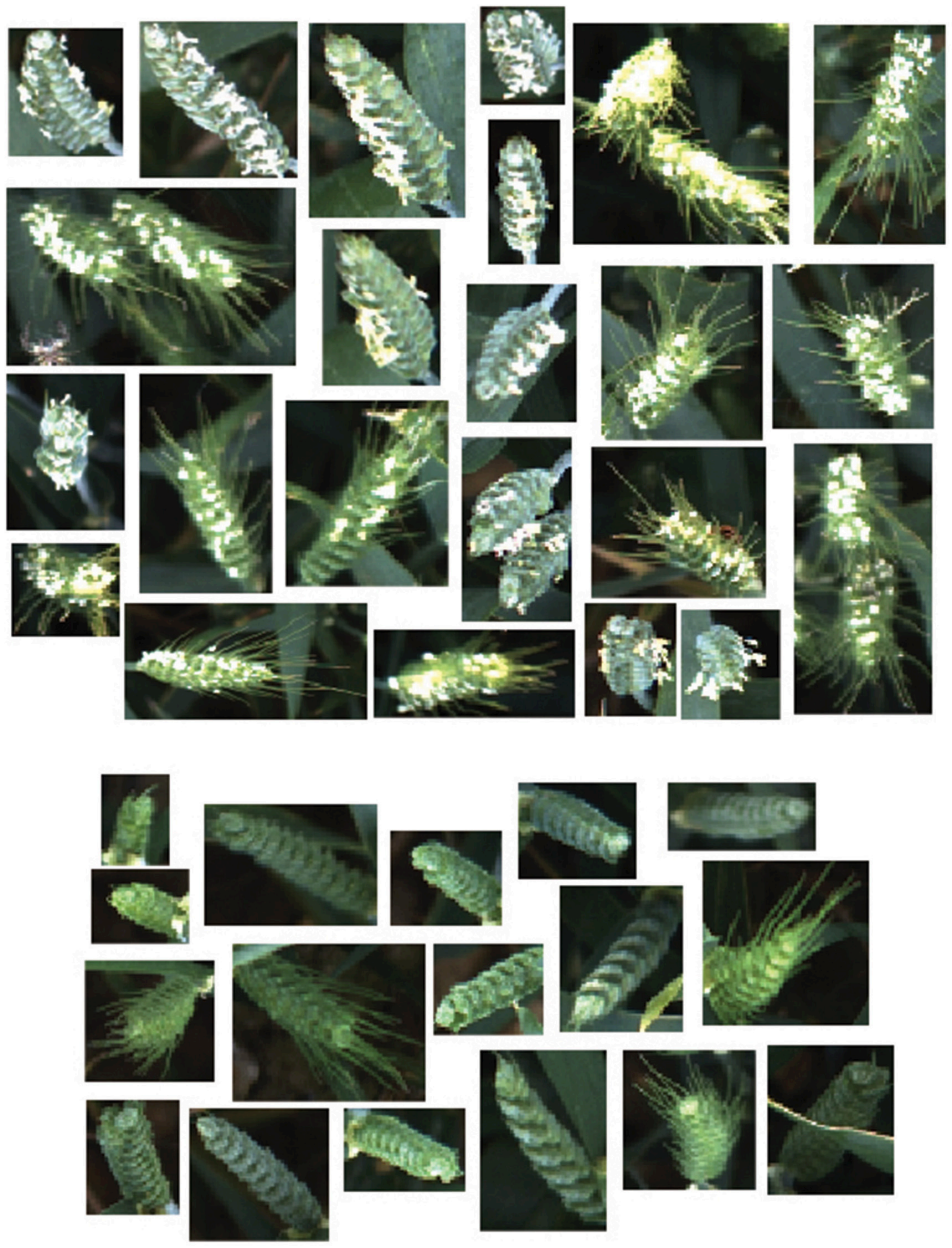
**Example of flowering training patches**. Examples of positive training patches of three different cultivars; (*Triticum aestivum* L. cv. Soissons, Maris Widgeon, and Cadenza), which contain flowering ears. Examples of background training patches which do not contain flowering ears.

Figure 11 shows randomly selected samples from the training patches. All training images were collected without considering the environmental changes and positioning or occlusion. As flowering development may be completed in only a few days, the beginning or intermediate stages can be easily missed. Therefore, all flowering images along the flowering duration were included. For the testing dataset, 108 full size canopy images were used with the original resolution, which includes 54 canopies with ears during flowering stage and 54 canopies with ears before or after flowering stage. The method selected to test the flowering stage was the one which produced the best result in the ear emergence experiment (decorrelation stretching, SIFT, LLC, and SPM algorithms with the vocabulary length of 2,000). The method was tested on each cultivar separately, as well as all three together. For all three cultivars, 38 images out of 54 images were retrieved correctly, which shows 82.54% accuracy. On the other hand, the accuracy when testing Soissons, Cadenza, and Widgeon individually was 76.72, 92.91, and 80.33%, respectively (Table 3).

**Table 3:** **Comparison of flowering accuracy between three wheat cultivars**.

**Wheat cultivar**	**No. training images**	**No. testing images**	**Accuracy (%)**
Cadenza	410	16	92.91
Soissons	410	23	76.72
Maris widgeon	410	15	80.33
All three	410	54	82.54

### 3.3. Discussion

To the best of our knowledge, few efforts have been made to automate the detection of crop growth stage (Thorp and Dierig, 2011; Yu et al., 2013; Guo et al., 2015; Zhu et al., 2016). Furthermore, the published methods have only been applied to small sections of the crops and generally tested only on a single cultivar. Unlike alternative methods, such as Yu et al. (2013), which used color properties to determine growth stages of maize, our approach uses rich feature collection techniques, such as SIFT, which carry suitable information to discriminate images at the category level on the canopy scale. The technique used by Guo et al. (2015) was only tested on two rice varieties individually at flowering stage and obtained just over 80% accuracy. However, our method integrated statistical variables, such as vector coding and spatial pyramid matching, which improved the accuracy and general versatility of the growth stage identification. On the other hand, their training system contained only flowering rice as the positive class and leaves as the negative class; failing to define rice before and after the flowering stage. This may have likely made their dataset more challenging because more variables would be added to the training dataset and distinguishing between non-flowering and flowering panicles would have added difficulty, potentially detecting false positives, ultimately reducing the accuracy of their method.

In our case, the accuracy of flowering detection is less than heading. This could be due to the size and color of anthers. The color of anthers can range from yellow to white depending on the cultivar, and the pale color of the anther has increased the sensitivity to over/under exposure as a result of changes in ambient illumination. Moreover, anthers are far smaller objects compared to wheat ears and are prone to noise, adding difficulty to detection them accurately. Nevertheless, the proposed method yielded greater accuracy than the existing method (Guo et al., 2015).

Pre-processing is also an important factor in our method. Newly emerging ears are difficult to distinguish as they are nearly the same color as the canopy, making methods based on color features inadequate for this purpose. However, the use of color enhancement methods, such as decorrelation stretching, yields higher accuracy. In our case, the absence of decorrelation stretching, results a decrease in accuracy from 95.24% to 57.29% and from 99.59 to 85.38% at earliest and latest stage of heading, respectively. Moreover, applying decorrelation stretching as a color enhancement tool early in the process minimize various ambient light conditions. The other important factor is the low level feature extraction in the BoVW process. SIFT was replaced by SURF as an alternative technique; however, although SURF performs faster as a result of using integral images and Hessian Matrix (Bay et al., 2006); SIFT still outperformed SURF (Table 2) in our experiment. It has also been examined that SIFT showed more stability on blurry images and more robust to rotation and scale invariants (Mikolajczyk and Schmid, 2005).

It should also be highlighted that the quality of the training dataset plays an important role in the overall performance. We aimed to define more scenarios for the system (e.g., ears at different positions, scales, and illumination conditions in the field, etc.). As shown in Figure 11, the accuracy of the ear emergence detection would increase by adding more training data. We would expect to improve the accuracy of the flowering experiment, by collecting data more frequently during the flowering period and increasing the size of the training dataset.

## 4. Conclusion

We proposed an automated observing system using computer vision to determine two key growth stages in wheat: ear emergence and flowering time. The proposed method is capable of distinguishing the critical growth stages from the RGB images taken in the field. The approach demonstrated a high performance for identifying such development changes and was not affected by the environmental conditions or illumination invariants in the field.

In future work, we aim to test our proposed method on additional wheat genetic material and other species, and in addition, to investigate the effect of alternative computer vision techniques from features extraction to classification on the performance and overall accuracy. Finally, we aim to apply the proposed method on images acquired by Unmanned Aerial Vehicles (UAVs) to monitor large fields efficiently and believe it will dramatically accelerate the recording of such development stages.

## Author contributions

PS proposed, developed, and tested the method; KS and NV planned and conducted the experiment; MH contributed to the revision of the manuscript and supervised the experiment; PS, KS, and NV contributed to writing the manuscript; all authors read and approved the final manuscript.

### Conflict of interest statement

The authors declare that the research was conducted in the absence of any commercial or financial relationships that could be construed as a potential conflict of interest.

## Abbreviations

BoVW: bag of visual words
SVM: support vector machine
SPM: spatial pyramid matching
RBF: radius basis kernel
LLC: local linear constraint
SIFT: scale invariant feature transform
DoG: difference-of-gaussian
LoG: laplacian-of-gaussian
PCA: principle component analysis
SURF: speeded up robust features
KNN: k-nearest-neighbors
UAV: unmanned aerial vehicle.
